# Identifying disease trajectories with predicate information from a knowledge graph

**DOI:** 10.1186/s13326-020-00228-8

**Published:** 2020-08-20

**Authors:** Wytze J. Vlietstra, Rein Vos, Marjan van den Akker, Erik M. van Mulligen, Jan A. Kors

**Affiliations:** 1 Department of Medical Informatics, Erasmus University Medical Center, Dr. Molewaterplein 50, 3015 GE Rotterdam, the Netherlands; 2 Department of Methodology & Statistics, Maastricht University, PO Box 616, 6200 MD Maastricht, the Netherlands; 3 Institute of General Practice, Johann Wolfgang Goethe University, Theodor-Stern-Kai 7, D-60590 Frankfurt, Germany; 4 Department of Family Medicine, Maastricht University, PO Box 616, 6200 MD Maastricht, the Netherlands

**Keywords:** Knowledge graph, Disease trajectories, Predicates, Temporal relationships, Directionality of predicates, Protein-protein interactions

## Abstract

**Background:**

Knowledge graphs can represent the contents of biomedical literature and databases as subject-predicate-object triples, thereby enabling comprehensive analyses that identify e.g. relationships between diseases. Some diseases are often diagnosed in patients in specific temporal sequences, which are referred to as disease trajectories. Here, we determine whether a sequence of two diseases forms a trajectory by leveraging the predicate information from paths between (disease) proteins in a knowledge graph. Furthermore, we determine the added value of directional information of predicates for this task. To do so, we create four feature sets, based on two methods for representing indirect paths, and both with and without directional information of predicates (i.e., which protein is considered subject and which object). The added value of the directional information of predicates is quantified by comparing the classification performance of the feature sets that include or exclude it.

**Results:**

Our method achieved a maximum area under the ROC curve of 89.8% and 74.5% when evaluated with two different reference sets. Use of directional information of predicates significantly improved performance by 6.5 and 2.0 percentage points respectively.

**Conclusions:**

Our work demonstrates that predicates between proteins can be used to identify disease trajectories. Using the directional information of predicates significantly improved performance over not using this information.

## Background

Knowledge graphs can be used to represent the biomedical knowledge published in literature and databases [1]. Knowledge is formalized as subject-predicate-object triples, where pairs of entities are related to each other by predicates [2]. By integrating triples from a variety of sources, knowledge graphs can be used to perform computational analyses on the comprehensive body of biomedical knowledge [3]. Previous work has used such analyses to identify new relationships between pairs of entities, e.g., between drugs and diseases [4, 5], genes and phenotypes [6, 7], or between diseases [8, 9].

Much research has been performed with knowledge graphs that only consist of proteins, commonly referred to as protein-protein interaction networks. Through the involvement of proteins in metabolic, signaling, immune, and gene-regulatory networks, protein-protein interaction networks can help to mechanistically explain disease and physiological processes [10–12]. Even though predicates further specify the types of interactions between proteins, thereby providing additional information that can be analyzed, protein-protein interaction networks usually do not use them. Instead, most methods analyze the network topology of proteins [12]. However, we have recently shown that analyses that are performed on protein knowledge graphs benefit from predicate information [13].

By using the predicates that specify the mechanisms by which proteins interact, temporal pathobiological relationships may also be identified, although this has not been demonstrated yet. A key application for such temporal analyses is the identification of disease trajectories, which are commonly occurring temporal sequences of diseases diagnosed in patients [14, 15]. An example of a disease trajectory found in a study by Jensen et al. [14] is *rheumatoid arthritis-precedes-heart failure*, where *precedes* is defined as “occurs earlier in time. […]” [16]. The occurrence of the reverse, *heart failure-precedes-rheumatoid arthritis*, was found to occur significantly less frequently in the same study, and therefore was not classified as a trajectory.

Identifying relationships between diseases is an important and popular research topic for protein-protein interaction networks (see Related work section). In such analyses diseases are represented by so-called disease proteins, which are proteins encoded by genes that are associated with a disease [17, 18]. Often cited benefits include an improved understanding of the biological mechanisms underlying disease interactions [8, 19, 20], and the ability to anticipate the next disease, thereby providing the knowledge necessary to improve treatment plans and interventions [14, 21]. However, the temporal aspects of relationships between diseases still require further investigation. We therefore aim to automatically determine whether a given sequence of two diseases forms a trajectory. We do so by leveraging the predicate information from paths between (disease) proteins in a knowledge graph. We also determine whether there is added value in using directional information of predicates for this task.

## Related work

Previous authors have mostly focused on identifying undirected relationships between diseases with protein networks [19–23]. For example, Kontou et al. created a disease-disease graph, where an edge between diseases indicated that they shared at least one disease gene [23]. Sun et al. calculated the similarity between diseases based on their shared disease proteins, shared physiological processes associated with these proteins, or the graph structures between the proteins [20]. Li and Agarwal identified which biological pathways were associated with diseases via their disease proteins, and identified relationships between diseases based on the number of shared pathways [19]. Menche et al. identified so-called disease modules, which are clusters of closely interrelated disease proteins [22]. They found that short distances between the modules of diseases were predictive for pathobiological relationships. Contrary to Kontou et al., they demonstrated that sharing disease proteins is not a requirement for diseases to be related to each other.

To our knowledge, Bang et al. were the only ones to use a directed protein-protein interaction network to identify disease trajectories [21]. The disease proteins of pairs of diseases were used to identify shared biomolecular pathways, after which the locations of the disease proteins within these pathways were determined. The disease with most upstream disease proteins was classified as the first within the sequence of diseases. Additionally, 13 million Medicare records were used to calculate two relative risk scores for each pair of diseases, corresponding with the two possible temporal sequences of the disease pair. If the sequence determined with the protein pathways concurred with the sequence that generated the largest relative risk, that sequence was identified as a trajectory. Between a total of 2604 diseases, their method suggested 61 trajectories. These were evaluated with the biomedical literature, where further leads were found for 16 of them. Because the authors only evaluated the trajectories that were suggested by their method, it is unclear how many trajectories the method failed to identify.

## Materials & methods

### Reference sets

The ability of our method to identify disease trajectories was evaluated with two reference sets, which have identified disease trajectories by different means. The first reference set consisted of statistically-derived disease trajectories from a large retrospective study of Danish hospital data, while the second set consisted of literature-validated disease trajectories that were based on a small prospective study of Dutch general-practitioner data.

#### Jensen reference set

The first reference set was based on a study of Jensen et al. [14]. They retrospectively identified 4014 disease trajectories from 6.2 million electronic patient records of Danish hospitals based on diagnoses assigned over 14.9 years. All diagnoses in these patient records were represented as International Statistical Classification of Diseases and Related Health Problems 10th Revision (ICD-10) codes. Jensen used the hierarchy within the ICD-10 to aggregate all diagnoses to a high abstraction level, resulting in 681 two-digit codes, such as “Malignant neoplasm of breast” (C50) or “Type 2 diabetes mellitus” (E11).

Jensen derived the disease trajectories from the Danish hospital data in a two-step process. First, they identified sequences of two diseases that were diagnosed within 5 years from each other in at least 10 patients, and which had a relative risk higher than 1. Subsequently, the direction of each sequence had to be corroborated by a binomial test that compared the frequency of the sequence to the frequency of its reversed sequence. Sequences that fulfilled both criteria were classified as disease trajectories.

To represent the diseases in the Jensen set on the protein level, we used the expert-annotated associations between proteins and diseases from the manually curated subset of DisGeNet [18]. The Unified Medical Language System (UMLS) MRCONSO table was used to map the ICD-10 codes of the Jensen trajectories to the UMLS identifiers that are used in DisGeNet. Two diseases, “Accidental poisoning by and exposure to other gases and vapours” (E47) and “Influenza due to identified zoonotic or pandemic influenza virus” (J09), were lost because their ICD-10 codes could not be mapped to a UMLS identifier. Because only 25% of the high-level diseases in the Jensen set were represented within DisGeNet, we used the “narrower” and “child” relationships from the UMLS MRREL table to identify subclasses of all diseases. By expanding the diseases with their subclasses, the percentage of diseases to which disease proteins could be assigned was increased to 68% (465 of 679 diseases).

From the 4014 disease trajectories in the Jensen set, there were 2530 trajectories where disease proteins could be assigned to both diseases (63%). These 2530 trajectories, which were used as positive cases in this reference set, contained 453 of the 465 diseases to which disease proteins could be assigned (97%). On average, diseases had 90 disease proteins assigned to them (median: 29, interquartile range: 7–94). Disease proteins were on average assigned to 6.2 diseases (median: 3, interquartile range: 2–8).

A set of 168,870 non-trajectories was constructed by creating all possible sequences of the 453 included diseases, minus the trajectories that were described by Jensen. The set of non-trajectories thereby included random pairs of diseases, the reversed temporal sequences of these random pairs, as well as the reversed temporal sequences of the trajectories. In the following, we will refer to the trajectories and non-trajectories as positive and negative cases to align with common terminology in the machine learning field.

#### Van den Akker reference set

The second reference set was based on a prospective cohort study on disease susceptibility by Van den Akker et al. [24]. They followed a Dutch cohort of 3460 patients over 2 years, during which their general practitioner notes were examined for sequences of International Classification of Primary Care (ICPC) codes that represent chronic, permanent, and recurrent diseases. In the Netherlands, each citizen is registered with a general practitioner, who acts like a gatekeeper for secondary and tertiary medical care, and is responsible for maintaining a complete medical history of the patient.

A total of 473 unique sequences of diseases were found in this cohort, containing 122 distinct diseases. Each sequence was manually evaluated using the published biomedical literature and medical handbooks. There were 65 sequences of diseases where the literature stated that the first disease increased the susceptibility of acquiring the second disease, and 408 sequences where no evidence of increased susceptibility was found. To maintain consistent terminology, we will refer to sequences with increased susceptibility as trajectories or positives and to sequences without increased susceptibility as non-trajectories or negatives.

To assign disease proteins to these 122 diseases we followed the same procedure as for the Jensen set by using the MRCONSO table to map the ICPC codes to UMLS identifiers, after which the MRREL table was used to group them with their subclasses. Disease proteins could be assigned to 97 diseases, which formed 55 trajectories and 316 non-trajectories. On average, diseases had 137 disease proteins assigned to them (median: 49, interquartile range: 17–167). Disease proteins were on average assigned to 3 diseases (median: 2, interquartile range: 1–4).

To determine whether our method could also identify the correct temporal sequence of the trajectories, 54 additional non-trajectories were created by reversing the sequence of the diseases in the literature-supported trajectories (the reverse sequence of one trajectory was already included as a non-trajectory in the data from the general practitioners).

### Knowledge graph

The predicates between proteins were extracted from the Euretos Knowledge Platform (EKP), a commercially available knowledge graph (http://www.euretos.com). In the EKP, information from more than 200 knowledge sources from a wide variety of domains in the life sciences is represented as triples. The biomedical entities such as proteins, drugs, or diseases that form the subjects and objects of these triples are represented in the knowledge graph as vertices, each of which has one or more identifiers associated with it from external databases. Mappings between the entities described in the different knowledge sources underlying the knowledge graph were made by matching their identifiers. The predicate and provenance of each triple are specified as part of an edge between the two vertices that represent the subject and object. The direction of the predicate goes from subject to object. The predicates in the underlying knowledge sources were matched to a standardized set of 203 predicates. If an exact match was not available, a predicate was manually mapped. If there were no explicit predicates in a database that was used as a knowledge source, the predicates were manually derived from the database schema. A path between two vertices is defined as a sequence of triples, or possibly a single triple, connecting the vertices.

The contents of the EKP are represented as documents in a NoSQL database, which allows them to be flexibly modelled and indexed. The EKP can be run on a reasonably-powered server, requiring an 8-core processor and 60GB of memory as a minimum. It has previously been used in pre-clinical research for drug efficacy screening [13], prioritizing existing drugs as repurposing candidates for autosomal dominant polycystic kidney disease [25], and pathway enrichment [26].

### Feature sets & machine learning

**Fig. 1:**
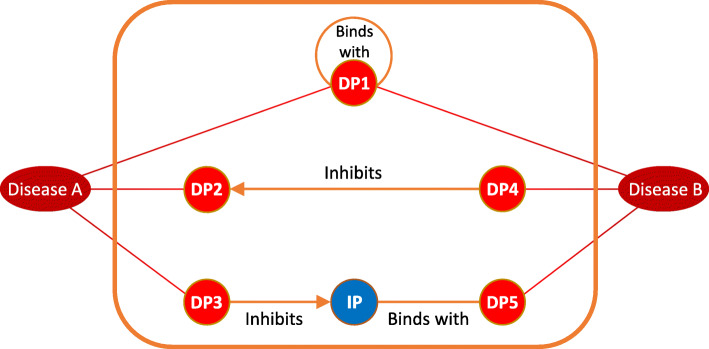
Schematic overview of the overlap, direct, and indirect scenarios that were extracted from the knowledge graph. Both diseases A and disease B have three disease proteins (DP) associated with them according to the manually curated subset of DisGeNet. DisGeNet describes that DP1 is known to be associated with both diseases, while the knowledge graph describes that it has a “binds with” relationship to itself. DP2 and DP4 have a direct “inhibits” relationship, and DP3 and DP5 are connected through an indirect path, by an intermediate protein (IP). The arrows between the proteins indicate which protein is the subject of the “inhibits” predicate, and which one its object. The “binds with” predicate was considered to be undirected by the experts, and therefore does not have a direction. Based on the paths in the knowledge graph, four feature sets are created, based on two methods to represent indirect paths, and both with and without the directional information of predicates

The paths between the disease proteins were extracted from the EKP. To keep our graph comprehensible, we only extracted paths that consisted of one or two triples, i.e., paths where two disease proteins are connected by at most one intermediate protein. Within this range, three scenarios for the paths between the disease proteins of two diseases A and B were distinguished (Fig. 1.):

1. Overlap, where A and B share a disease protein, optionally with a path to itself, e.g. a disease protein of which two copies bind with each other (homodimerization).
2. Direct path, where a disease protein of A and a disease protein of B are part of one triple.
3. Indirect path, where one intermediate protein connects the disease proteins of A and B, requiring a sequence of two triples.

Two different methods to represent indirect paths between disease proteins were compared. The first method constructed so-called metapaths [5], where the sequence of predicates in an indirect path was used as single feature. The second method, which we refer to as split paths, considered each predicate in the indirect paths as a separate feature [13]. Each method was tested both with and without directional information of predicates. Predicates were either considered to all be undirected, or predicates were categorized as being directed or undirected based on expert assessment (described in the Assessment of predicate directionality section below), which we refer to as the Mixed variation. In the overlap scenario, where the subject and the object were the same protein, predicates were always considered to be undirected.

**Fig. 2:**
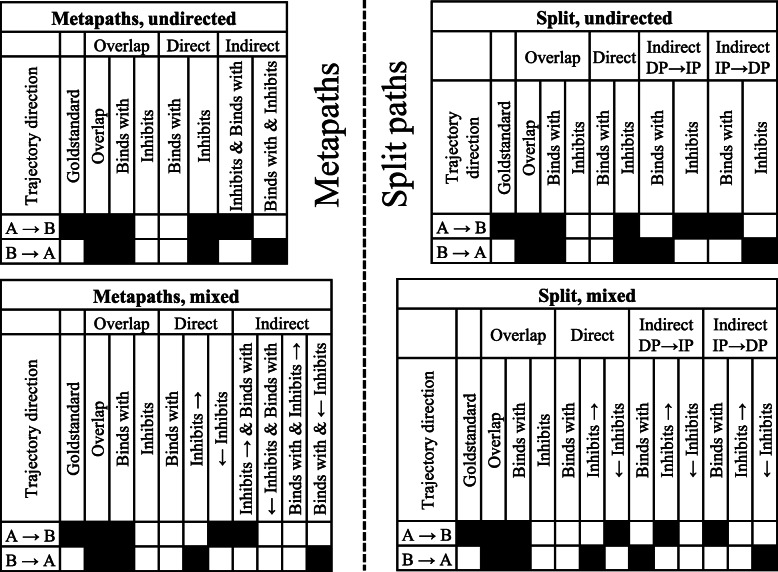
The four feature sets that were derived from the paths between the disease proteins in Fig. 1. All features are binary: Black fields indicate a “True” value, while empty fields indicate a “False” value. For the “Mixed” feature sets, the “Binds with” predicate is assessed to be undirected by experts, while the “Inhibits” predicate is assessed to be directed

All features were binary. Figure 2 shows the four feature sets that are derived from the example shown in Fig. 1. We also experimented with feature sets where all predicates were directed as indicated by the subject and object of the triple in the EKP. However, because some predicates are explicitly defined as being undirected, using any directional information from triples with these predicates would contradict these definitions. Nonetheless, for the sake of completeness we have chosen to present these results in Additional file 1.

Random forests were trained to classify the sequences of diseases as positive or negative. Classification performance was measured with the area under the receiver operator characteristic curve (AUC) of a 10-fold cross-validation experiment [27, 28]. We report the mean and standard deviation of the AUCs of 10 repeated cross-validation experiments. The same folds that were used in the experiments with undirected predicates were also used in the experiments with directed predicates, after which the differences between the test folds were tested for significance with a two-sided, paired t-test.

To control for the differences in prevalence and number of cases between the two reference sets, we also report the classification performance after undersampling the number of positive and negative cases in the Jensen set to match those in the Van den Akker set.

For the best performing classifiers we also report sensitivity and specificity at the probability cutoff for which the Youden index (sensitivity + specificity – 1) is largest [29].

Machine learning and evaluation of results were performed in R [30] with the packages caret [31], ranger [32], and pROC [33].

### Assessment of predicate directionality

Three experts with a strong biomedical background and familiarity with knowledge graphs assessed the directionality of 47 distinct predicates that were found in the extracted paths. They were provided with definitions of these predicates which were obtained from the Pathway Commons resource [34]. If not available, definitions were sought through the National Library of Medicine [35], or the OBO foundry [36]. The assessors could categorize each predicate as “directed”, “undirected”, or “don’t know”. To establish directionality, a predicate had to be categorized as directed or undirected by a majority (i.e., two or three) of the assessors. Predicates that contain a negation (e.g., “does not interact with”) were automatically categorized the same as the corresponding predicate without negation (“interacts with”), and therefore not presented to the assessors. For some predicates the categorization was straightforward. For example, Pathway Commons defines the predicate “interacts with” as “This is an undirected relation between participant proteins of a molecular interaction. […]” , and the predicate “catalysis precedes” as “This relation defines directed interactions between proteins. […]” [34]. Six predicates did not reach a majority in the first round and were anonymously commented upon by the assessors to motivate their categorization. These comments were shared between the assessors, after which they could update their initial choice. Each predicate was then categorized with a majority.

Table 1 shows the 12 predicates that were categorized as undirected by the three experts. The other 35 predicates were categorized as directed. A complete overview of the predicates can be found in Additional file 2.

**Table 1:** Predicates categorized as undirected as a result of the assessment process

Undirected Predicates	
binds with	
coexists with	
does not coexist with	
forms protein complex with	
interacts with	
does not interact with	
is associated with	
is compared with	
is functionally related to	
is spatially related to	
is the same as	
ortholog is associated with	

## Results

### Extracted paths

In total, 6859 distinct disease proteins were assigned to the diseases in both reference sets, three of which could not be mapped to the EKP. Another 430 (6.3%) of the disease proteins were not found in any of the extracted paths. From these disease proteins, 314 had no relationship to any other protein in the EKP.

The remaining 6426 disease proteins were involved in 1,338,310 direct paths and 833,546,575 indirect paths, while 2581 disease proteins had 7354 paths to themselves. All paths were based on 2,015,738 distinct triples, which contained 17,132 different proteins and 47 different predicates.

The overlap scenario, where the two diseases in the trajectory share at least one disease protein (scenario 1, Feature sets & Machine learning section), occurred in 58% of the positive cases of the Jensen set, and 95% of the positive cases of the Van den Akker set. No indirect paths (scenario 3, Feature sets & Machine learning section) were found between the disease proteins of 119 positive cases (4.7%), and 18,217 negative cases of the Jensen set (10.8%), and one positive case (1.8%) and 15 negative cases (4.1%) of the Van den Akker set.

### Classification results

The classification performance for both reference sets is shown in Table 2. Mixed metapaths performed best, achieving mean AUCs of 89.8% for the Jensen set and 74.5% for the Van den Akker set. Overall, classification performance on the Van den Akker set was 9.9 to 15.3 percentage points lower than on the Jensen set, while standard deviations were 9.6 to 11.3 percentage points higher. Metapaths performed 4.1 to 7.0 percentage points better than split paths. The performance of the mixed feature sets was 1.9 to 6.5 percentage points higher than the undirected feature sets. All differences between mixed and undirected feature sets were significant (*p*-values for Jensen metapaths and split paths: < 0.001; Van den Akker metapaths: 0.02, split paths 0.001).

**Table 2:** Classification results for the four feature sets for both reference sets

	Jensen set		Jensen set - undersampled		Van den Akker set	
	Metapaths	Split paths	Metapaths	Split paths	Metapaths	Split paths
Undirected	83.3 (1.7)	78.3 (1.7)	64.2 (12.1)	61.9 (12.3)	72.5 (11.8)	68.4 (13.0)
Mixed	89.8 (0.9)	82.8 (1.2)	82.3 (8.4)	69.6 (13.1)	74.5 (10.5)	70.3 (11.4)

The values in the columns indicate the mean AUC and its standard deviation in % of 10 cross-validation experiments

To quantify how much of the difference in AUC between the two reference sets could be attributed to their difference in size, the Jensen set was undersampled to the same number of positive and negative cases as the Van den Akker set. With the exception of the mixed metapaths, performance dropped below the performance that was achieved with the Van den Akker set. The standard deviations also increased from 0.9–1.7% to 8.4–12.3%. The latter values are comparable to the standard deviations on the Van den Akker set.

**Fig. 3:**
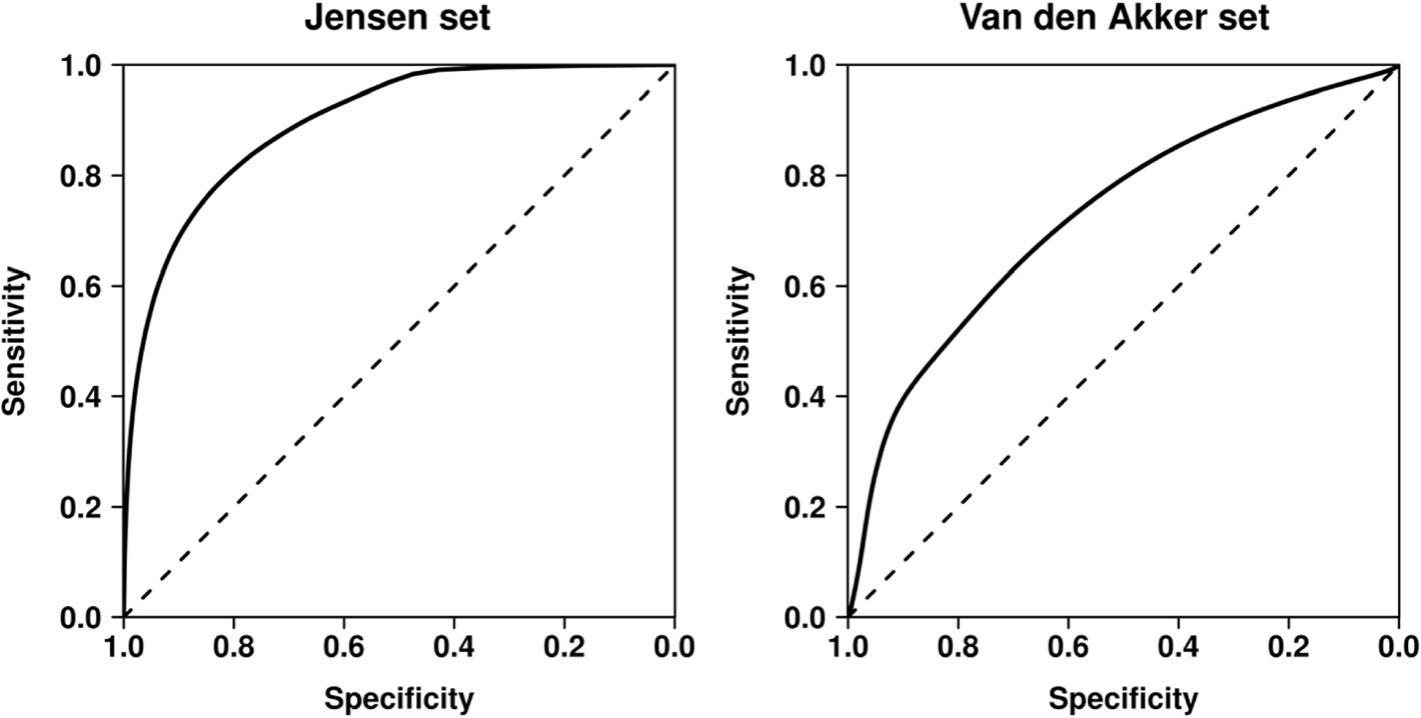
ROC curves of the mixed metapaths classifiers for the Jensen set and the Van den Akker set

Figure 3 shows the receiver operating characteristic (ROC) curves of the mixed metapath classifiers that performed best. For the Jensen set, sensitivity and specificity at the maximum Youden index were 79.2% and 82.4%, respectively, while for the Van den Akker set these were 73.6% and 64.3%.

### Error analysis

For our best classifier (mixed metapath features, trained on the Jensen set), we analyzed the top-15 false-positive and the top-15 false-negative cases, searching the literature for information that might explain the errors. The results of our analysis of the false positives are shown in Table 3. Overall, the first 10 out of the top 15 false positives appear to be omissions from the Jensen set rather than misclassifications. For two false-positive cases, potential mechanisms have been suggested, but the current evidence is inconclusive on whether those mechanisms are valid. For the remaining three false-positive cases no literature could be found, which may therefore be interesting leads for further investigation.

**Table 3:** Assessment of the top 15 false-positive trajectories

First disease	ICD-10	Second disease	ICD-10	Assessment
Mental and behavioural disorders due to use of alcohol	F10	Alzheimer’s disease	G30	Described in literature [37]
Essential (primary) Hypertension	I10	Alzheimer’s disease	G30	Described in literature [38]
Osteoporosis without pathological fracture	M81	Alzheimer’s disease	G30	Described in literature [39]
Non-insulin-dependent diabetes mellitus	E11	Alzheimer’s disease	G30	Described in literature [40]
Other disorders of pancreatic internal secretion	E16	Alzheimer’s disease	G30	Described in literature [41]
Schizophrenia	F20	Other septicaemia	A41	Described in literature, but commonly occurs via intermediate diseases such as agranulocytosis and pneumonia [42]
Lupus erythematosus	L93	Other disorders of urinary system	N39	Described in literature [43]
Disorders of vestibular function	H81	Alzheimer’s disease	G30	Described in literature [44]
Lupus erythematosus	L93	Respiratory failure, not elsewhere classified	J96	Described in literature [45]
Unspecified Dementia	F03	Dementia in Alzheimer’s Disease	F00	Further specification of diagnosis
Retinal vascular occlusions	H34	Cystitis	N30	No relationship found in literature
Chronic ischaemic heart disease	I25	Other septicaemia	A41	Cardiac troponins are suggested to be biomarkers for sepsis [46]
Hyperplasia of prostate	N40	Alzheimer’s disease	G30	No relationship found in literature
Hyperparathyroidism and other disorders of parathyroid gland	E21	Alzheimer’s disease	G30	Suggested in literature (via calcium) [47]
Asthma	J45	Umbilical hernia	K42	No relationship found in literature

Table 4 shows the results for the top-15 false negatives. For six false negatives, the second disease was likely to be caused by the treatment of the first disease. For example, the radiation that is used to treat the malignant neoplasm of the larynx may compromise the immune system around the throat and mouth, thereby increasing susceptibility to oropharyngeal candidiasis [54]. Two false-negative trajectories are likely to have mechanical causes, rather than molecular pathways. The trajectory from malignant neoplasms of the ovary to nutrient deficiency can be explained by the blocking of the intestines by the ovarian tumor, thereby blocking the entire digestive system [53]. For four of the false-negative trajectories, no description could be found in the literature, making their assessment impossible. Assessment of the three remaining false negatives is speculative. For example, the trajectory from transient ischemic attacks (TIA) to vitamin B12 deficiencies may be an artifact of the medical record keeping. Vitamin B12 is known to protect against TIAs [52], so what may often happen is that a vitamin B12 deficiency is only diagnosed after the more acute TIA has been treated in an emergency room.

**Table 4:** Assessment of the top 15 false-negative trajectories

First disease	ICD-10	Second disease	ICD-10	Assessment
Thyrotoxicosis [hyperthyroidism]	E05	Other disorders of eye and adnexa	H57	Likely side effect of treatment [48]
Irritable bowel syndrome	K58	Spondylosis	M47	No relationship found in literature
Vitamin B12 deficiency anaemia	D51	Other septicaemia	A41	Vitamin B12 has been hypothesized as treatment for sepsis [49]
Mental and behavioural disorders due to use of alcohol	F10	Acute and transient psychotic disorders	F23	Described in literature, but no clear role for protein interactions [50]
Gonarthrosis [arthrosis of knee]	M17	Erysipelas	A46	No relationship found in literature
Senile cataract	H25	Other disorders of lens	H27	Likely side effect of treatment [51]
Transient cerebral ischaemic attacks and related syndromes	G45	Vitamin B12 deficiency anaemia	D51	Only reverse described in literature, that vitamin B12 protects against stroke [52]
Malignant neoplasm of ovary	C56	Deficiency of other nutrient elements	E61	Likely mechanical cause [53]
Malignant neoplasm of larynx	C32	Candidiasis	B37	Likely side effect of treatment [54]
Other intervertebral disc disorders	M51	Somatoform disorders	F45	No relationship found in literature
Gonarthrosis [arthrosis of knee]	M17	Other local infections of skin and subcutaneous tissue	L08	No relationship found in literature
Benign neoplasm of brain and other parts of central nervous system	D33	Other septicaemia	A41	Likely intermediate through infection, which follows surgery or weakening of the immune system after (radiation) treatment
Insulin-dependent diabetes mellitus	E10	Other disorders of eye and adnexa	H57	Diabetes is a risk factor for many eye diseases [55], but it is not clear whether these fall under this ICD-10 code
Noninflammatory disorders of ovary, fallopian tube and broad ligament	N83	Ventral hernia	K43	Likely side effect of treatment [56]
Other intervertebral disc disorders	M51	Other polyneuropathies	G62	Likely mechanical cause [57]

## Discussion

Our work demonstrates that disease trajectories can be identified with the predicates between proteins in a knowledge graph. To do so, our machine-learning based methodology needed to successfully identify both the correct pairs of diseases, as well as their correct temporal sequences. Overall, representing indirect paths as metapaths performed superior as compared to representing them as split paths. Using the directional information of predicates significantly improved performance over not using this information. Undersampling the Jensen set to the same number of positive and negative cases as the Van den Akker set showed that its lower performance and higher standard deviations could partially be explained by its small size.

In previous work, Bang et al. [21] identified disease trajectories by calculating the relative risk between two diseases and combining this with the relative position of disease proteins in biomolecular pathways. Their method is fully dependent on shared disease proteins between the two diseases, whereas our method also works when there are only (in) direct paths between disease proteins. In the Jensen set, this holds for 42% of the trajectories. Performance comparison of the methods is difficult because Bang et al. only validated the disease trajectories that were suggested by their method, but did not validate the non-trajectories. Thus, only the precision of their method can be calculated but no insight is provided in the number of false-negative trajectories. A final complication for the comparison between the two methods is the claim of Bang et al. to discover causal relationships between diseases, rather than only temporal ones. Unfortunately, they refer to an example to define causal relationships between diseases, making it difficult to pinpoint how these differ from disease trajectories.

Although we do not foresee direct clinical application of our work, our high performance may persuade experts to further examine the protein paths underlying some positively classified trajectories, either known or newly suggested. Interpreting these protein paths might provide additional clues about the mechanism through which the first disease leads to the second. Identifying and understanding these mechanisms is likely to improve prevention, prediction of disease progression, intervention, and drug development, thereby indirectly supporting clinical practice and health-care policy. For now, such detailed examinations of the protein paths were beyond the scope of this project.

A downside of working on the protein level was that not all disease trajectories could be studied. More than a third of the trajectories of the Jensen set, and a fifth of the Van den Akker set was lost because disease proteins could not be assigned to one or both of the diseases in a trajectory. Even when disease proteins could be assigned to both diseases, alternative explanations were sometimes more plausible. For example, our analysis of the false-negative cases suggested that some trajectories could be explained mechanically, or were likely due to a side effect of the treatment for the first disease. To determine the true performance of our method, a validated set of trajectories that are caused by biomolecular mechanisms would be needed. Alternatively, the range of trajectories that can be analyzed may be broadened by linking diseases to other types of disease information available in the EKP, e.g., information about drugs or physiological processes.

The two reference sets that were used in this research were both based on patient data, but differed in many other respects. The sequences of diseases in the Jensen set were classified as trajectories based on statistics calculated from 15 years of nationwide hospital data. Despite this large volume of data, our analysis of the false-positive cases showed that the set of trajectories was incomplete. The literature evaluation underlying the Van den Akker set ensures that such omissions are unlikely to occur there. Furthermore, the negatives in the Van den Akker set either were observed in patients, or were reversals of literature-supported trajectories. Because the negative cases in the Jensen set were based on randomization, this set is likely to contain pairs of diseases that never co-occur within patients. Finally, the types of diagnoses within the trajectories differ between the two reference sets. The hospital patients in the Jensen set are more likely to suffer from more serious and complicated diseases than patients visiting a general practitioner in the Van den Akker set. On the other hand, the Van den Akker set only included chronic, permanent, and recurring diseases, thereby excluding diseases that are acute and rapidly treatable.

Only the definitions from Pathway Commons stated whether the predicate was directed or not. The definitions of predicates from other knowledge sources, including the National Library of Medicine, left room for interpretation. As a result, six predicates required a second round of assessment before a majority was achieved between the assessors. With ontologies playing increasingly important roles in data standardization and sharing [58], the directionality of predicates should always be clear. The Relationship Ontology already supports categorization of predicates as directed or undirected, which it refers to as asymmetric or symmetric predicates, but unfortunately is far from complete and did not cover the predicates in our set [59].

A potential new application for our method would be to identify trajectories for rare and low-prevalence diseases, where insufficient patient data is available for studies such as those performed by Jensen or Van den Akker. Because our method identifies trajectories based on a protein network, it is independent of the prevalence of a disease. Furthermore, many of the estimated 5 to 8 thousand rare diseases have well known genetic causes [60], making them highly suitable to be analyzed with our method.

A possible extension of our work would be the identification of longer disease trajectories, e.g. the trajectories consisting of sequences of four diseases that were also described by Jensen et al. [14]. However, as far as we are aware all available knowledge-graph methods limit themselves to identifying relationships between two entities. Expanding the current methods to identify longer sequences should therefore be a separate investigation.

## Conclusions

Our work demonstrates that disease trajectories can be identified with the predicate information from a knowledge graph. We also demonstrate and quantify the added value of using directional information of predicates for this task. Our work thereby enables the discovery of temporal relationships with predicate information from knowledge graphs.

## Supplementary information

**Additional file 1. **Description and results of the directed variation feature sets. This file describes the feature sets and classification results of the variation where all predicates in the feature sets have a direction as specified by their triples in the knowledge graph. Their categorization as directed or undirected by the assessors was not used in this variation. Figure S1 shows an example of the feature sets derived from Fig. 1, with the difference that in this variation the “Binds with” predicate also is directed. Table S1 shows the classification performance of the directed feature sets along with the performances of the undirected and the mixed variations. Table S2 shows the *p*-values of the two-sided paired t-tests between all variations.

**Additional file 2.** Overview of predicates that were found in the paths. This file contains Table S3, which shows the 47 predicates that connect proteins in the knowledge graph and were used to construct the features.

## Data Availability

The datasets and scripts that are used in this study are available at https://github.com/Wytz/DiseaseTrajectories

## Abbreviations

AUC: Area under the receiver operator characteristic curve
DP: Disease Protein
EKP: Euretos Knowledge Platform
ICD-10: International Statistical Classification of Diseases and Related Health Problems 10th Revision
ICPC: International Classification of Primary Care
IP: Intermediate Protein
ROC: Receiver Operating Characteristic curve
TIA: Transient Ischemic Attack
UMLS: Unified Medical Language System
