# Supplementary material for: Identifying disease trajectories with predicate information from a knowledge graph
Source: J Biomed Semantics. 2020 Aug 20;11:9. doi: 10.1186/s13326-020-00228-8 (PMC7439632; doi:10.1186/s13326-020-00228-8)
Supplement: Supplementary file 1 — Additional file 1. Description and results of the directed variation feature sets. This file describes the feature sets and classification results of the variation where all predicates in the feature sets have a direction as specified by their triples in the knowledge graph. Their categorization as directed or undirected by the assessors was not used in this variation. Figure S1 shows an example of the feature sets derived from Fig. 1, with the difference that in this variation the “Binds with” predicate also is directed. Table S1 shows the classification performance of the directed feature sets along with the performances of the undirected and the mixed variations. Table S2 shows the p-values of the two-sided paired t-tests between all variations. [file 13326_2020_228_MOESM1_ESM.docx]

Feature sets where all predicates are directed

In addition to the undirected and the mixed feature sets, we also experimented with feature sets where all predicates were considered to be directed, as indicated by the subject and object of the triple. Figure 1 shows example feature sets for both the metapaths and the split paths.

Figure S1 Examples of the directed feature sets for both the metapaths and the split paths. These features were derived from the same knowledge graph as shown in Figure 1 in the manuscript. Because all predicates in this variation are directed, the “binds with” predicate is also directed in accordance with the subject and the object of the triple. Directionality of triples is only included for the direct and indirect scenarios, and not for the overlap scenario.

The classification performance for both reference sets, and their six possible feature sets is shown in Table 1. As can be seen, the directed feature sets achieved almost identical performance to the mixed feature sets.

Table S1 Classification performance results based on 10 repeats of a ten-fold cross validation experiment.

|  | Jensen reference set | | Van den Akker reference set | |
| --- | --- | --- | --- | --- |
|  | Metapaths | Split paths | Metapaths | Split paths |
| Undirected | 83.3 (1.7) | 78.3 (1.7) | 72.5 (11.8) | 68.4 (13.0) |
| Mixed | 89.8 (0.9) | 82.8 (1.2) | 74.5 (10.5) | 70.3 (11.4) |
| Directed | 90.0 (0.8) | 83.1 (1.4) | 74.1 (10.3) | 70.8 (11.5) |

To determine whether the differences in AUC between the mixed and directed feature sets was statistically significant, we again performed paired T-tests by sampling identical folds between experiments. The differences between the mixed and directed feature sets for both reference sets were not significant. See Table 2 for a complete overview of all p-values.

Table S2 p-values of the paired t-tests of the AUCs of the different cross-validation experiments.

|  | Jensen set | | Van den Akker set | |
| --- | --- | --- | --- | --- |
|  | Metapaths | Split paths | Metapaths | Split paths |
| Undirected – Mixed | <0.001 | <0.001 | 0.022 | 0.001 |
| Mixed - Directed | 0.107 | 0.882 | 0.872 | 0.318 |
| Undirected - Directed | <0.001 | <0.001 | 0.488 | <0.001 |
