# Supplementary material for: Identifying disease trajectories with predicate information from a knowledge graph
Source: J Biomed Semantics. 2020 Aug 20;11:9. doi: 10.1186/s13326-020-00228-8 (PMC7439632; doi:10.1186/s13326-020-00228-8)
Supplement: Supplementary file 2 — Additional file 2. Overview of predicates that were found in the paths. This file contains Table S3, which shows the 47 predicates that connect proteins in the knowledge graph and were used to construct the features. [file 13326_2020_228_MOESM2_ESM.docx]

Overview of predicates found in paths

Table S3 Overview of all 47 predicates that were found in the paths between the proteins in the EKP.

| affects | is a |
| --- | --- |
| augments | is associated with |
| binds with | is compared with |
| catalysis precedes | is functionally related to |
| coexists with | is higher than |
| consumption controlled by | is lower than |
| controls expression of | is manifestation of |
| controls phosphorylation of | is not higher than |
| controls state change of | is not lower than |
| controls transport of | is not part of |
| converts to | is parent of |
| disrupts | is part of |
| does not coexist with | is spatially related to |
| does not convert to | is the same as |
| does not inhibit | is tributary of |
| does not interact with | is variant of |
| does not produce | manages |
| does not stimulate | ortholog is associated with |
| forms protein complex with | performs |
| gene product is biomarker type | produces |
| gene product is encoded by | stimulates |
| gene product variant causes | targets |
| inhibits | uses |
| interacts with |  |
